# Composition of incubation solution impacts in vitro protein uptake to silicone hydrogel contact lenses

**Published:** 2012-02-04

**Authors:** Salsabeel Jadi, Miriam Heynen, Doerte Luensmann, Lyndon Jones

**Affiliations:** Centre for Contact Lens Research, School of Optometry, University of Waterloo, Waterloo, Ontario, Canada N2L 3G1

## Abstract

**Purpose:**

To determine the impact of incubation solution composition on protein deposition to silicone hydrogel (SH) contact lenses using a simplistic and a complex model of the tear film.

**Methods:**

Three SH materials – senofilcon A (SA), lotrafilcon B (LB), and balafilcon A (BA) – were incubated in two different solutions; Solution A was a simplistic augmented buffered saline solution containing a single protein, whereas Solution B was a complex artificial tear solution (ATS), containing the augmented buffered saline solution in addition to proteins, lipids, and mucins (pH=7.4). The proteins of interest (lysozyme, lactoferrin, albumin) were radiolabeled with Iodine-125 (2% protein of interest) and the accumulation of the conjugated protein to the lens materials was determined after 1, 7, 14, and 28 days of incubation. Protein deposition was measured using a gamma counter and the raw data were translated into absolute amounts (µg/lens) via extrapolation from standards.

**Results:**

After 28 days, lysozyme uptake was significantly lower on BA lenses when incubated in Solution A (33.7 μg) compared to Solution B (56.2 μg), p<0.001. SA lenses deposited similar amounts of lysozyme when incubated in either Solution A (2.6 μg) or Solution B (4.1 μg), p>0.05. LB lenses also deposited similar amounts of lysozyme for both solutions (Solution A: 5.0 μg, Solution B: 4.7 μg, p>0.05). After 28 days, BA lenses accumulated approximately twice the amount of lactoferrin than the other lens materials, with 30.3 μg depositing when exposed to Solution A and 22.0 μg with Solution B. The difference between the two solutions was statistically significant (p<0.001). LB materials deposited significantly greater amounts of lactoferrin when incubated in Solution A (16.6 μg) compared to Solution B (10.3 μg), p<0.001. Similar amounts of lactoferrin were accumulated onto SA lenses regardless of incubation solution composition (Solution A: 8.2 μg, Solution B: 11.2 μg, p>0.05). After 28 days, albumin deposition onto BA lenses was significantly greater when lenses were incubated in Solution B (1.7 μg) compared to Solution A (0.9 μg), p<0.001. Similar amounts of albumin were deposited on SA lenses when incubated in either solution (0.6 μg versus 0.7 μg, p>0.05). LB lenses incubated in Solution A deposited more albumin compared to Solution B (0.9 μg versus 0.6 μg), p=0.003.

**Discussion:**

Protein deposition onto SH materials varied when contact lenses were incubated in either a complex ATS compared to a single protein solution. More lysozyme accumulated onto BA lenses incubated in a complex analog of the human tear film, whereas lactoferrin deposited onto SA lenses independent of incubation solution composition. To better mimic the ex vivo environment, future studies should use more appropriate analogs of the tear film.

## Introduction

Silicone hydrogel (SH) lenses became increasingly popular over the last decade primarily due to their higher oxygen permeability, leading to reduced hypoxic complications compared to poly-2-hydroxyethyl methacrylate (pHEMA)-based lenses [1,2]. A recent survey indicated that 54% of all contact lens wearers in the United States (US) were fitted with SH materials for daily wear, as compared with only 15% using hydrogel lenses [3]. This has changed greatly since 2005, where only 22% of the lens wearers in the US were fitted with SH lenses [4].

Contact lenses are prone to protein deposition, the amounts of which are dependent on the chemical composition of the lens materials [5,6]. Several studies have shown that deposition onto contact lenses may cause discomfort [7] acute red eye [8], and inflammatory reactions [9]. Deposited proteins denature over time and hence may cause inflammatory responses to the palpebral conjunctiva, such as giant papillary conjunctivitis [10]. Contact lens wear can lead to microbial keratitis through infection of the cornea by pathogenic organisms, such as gram-negative *Pseudomonas aeruginosa*, which adhere to the protein-coated lens material [11]. Tear film deposits may further reduce visual acuity [12] and surface wettability [13].

Several different tear film proteins have been detected in the proteomic profiles deposited on SH contact lenses, including albumin, lipocalin, lactoferrin, and lysozyme [14,15]. Many other proteins have further been extracted from worn contact lenses, some examples are complement C3 [16], immunoglobulin E (IgE) [17], immunoglobulin G (IgG) [18], and secretory phospholipase A2 [19]. Using antibody arrays, several chemokines, cytokines, and growth factors have been detected in the human tear film [20], as well as proteases and protease inhibitors detected through mass spectrometers [21]. There are more than 100 different proteins identified in the tear film [21,22], constituting a protein concentration of around 8 mg/ml [21,23]. Lysozyme is primarily used as the “model protein” for in vitro studies investigating deposition on lenses. The main reasons for this are the high abundance of this positively charged protein in the tear film and the fact that it accounts for approximately 90% of the deposited protein on ionic (negatively charged) pHEMA-based lenses [16,24].

Most SH contact lenses available today are non-ionic and deposit substantially less protein than ionic conventional hydrogels [25]. Deposition profiles are often determined using simplified in vitro models, however, there are several differences between in vitro and ex vivo results when comparing protein accumulation on contact lenses [14,26-28]. The in vitro model typically lacks the effect of blinking, surface drying, the cleansing process of contact lenses between hours of wear, and the physiologic events that are naturally occurring in the eye. As a result, the level of lysozyme deposition determined on ionic pHEMA lenses is typically slightly lower on worn lenses compared to data collected on in vitro deposited lenses (ex vivo=985–991 μg of lysozyme [26,29], in vitro=1,434–1,800 μg of lysozyme [27,30,31]). In comparison to pHEMA, SH lenses deposit much lower amounts of lysozyme, averaging <20 μg/lens [26,32]. SH materials generally accumulate similar amounts of protein, except for the ionic SH material balafilcon A (BA), which deposits much great amounts of protein per lens [5,30]. Subbaraman and colleagues illustrated in an in vitro study that senofilcon A (SA) and lotrafilcon B (LB) lenses deposited 3.7 μg and 6.1 μg of lysozyme, whereas BA deposited more than three times that amount (19.4 μg) after two weeks of incubation [30]. Ex vivo data from Subbaraman et al. [33] have further shown that after two weeks of lens-wear, SA and LB deposit similar amounts of total protein - 4.6 μg and 6.6 μg, respectively, - whereas BA deposits 26.9 μg, which is only marginally higher compared to the lysozyme in vitro results. Zhao and colleagues demonstrated a similar pattern, where BA lenses deposited the greatest amount of protein and SA the least; however, SA lenses deposited significantly less protein (0.1 μg [5]) than findings by Subbaraman and colleagues (4.6 μg [33]).

In vitro deposition studies have limitations when single protein solutions are used, as they cannot accurately mimic the ocular tear film, due to their lack of other tear film components, including other proteins, lipids and mucins [30]. The use of more complex artificial tear solutions (ATS) on pHEMA-based contact lenses has shown to impact lipid and lysozyme uptake onto the lens material [34-36]. Whether proteins that are different in size and charge respond in a similar fashion when depositing to SH lenses is not clear, therefore the purpose of this in vitro study was to compare the amount of protein uptake on different SH lens materials using two different in vitro models. The first model uses an augmented buffered saline solution with a single protein added, whereas the second model uses a far more complex ATS, consisting of the augmented buffered saline solution as its base, for lens incubation.

## Methods

A single protein solution and a complex ATS were used to investigate potential differences in protein deposition to SH materials, using radiolabeled lysozyme, lactoferrin and albumin.

Three SH contact lens materials were investigated in this study, senofilcon A (SA, ACUVUE OASYS; Johnson & Johnson, Jacksonville, FL), lotrafilcon B (LB, Air Optix; CIBA VISION, Duluth, GA), and balafilcon A (BA, PureVision; Bausch & Lomb, Rochester, NY). These lenses have been categorized in different Food and Drug Administration (FDA) groups, with both SA and LB belonging to FDA group I (low water content <50%, non-ionic), whereas BA belongs to FDA group III (low water content <50%, ionic).

**Figure 1:**
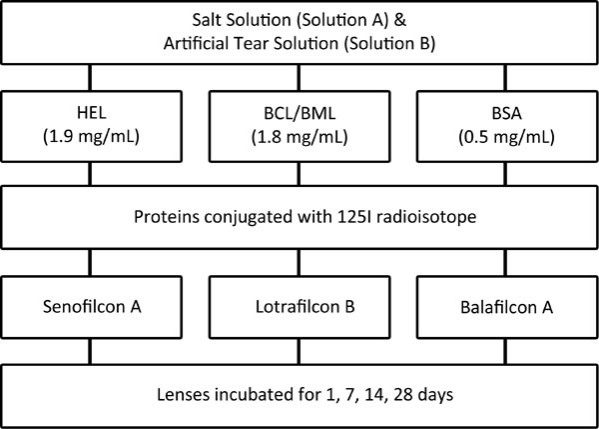
Flowchart depicting layout of two-part study, where each time point contained three replicates.

Two independent studies were performed in parallel to investigate the deposition of a single protein when added to a saline solution compared to a complex ATS (Figure 1). To identify the protein of interest in the solution and on the lens, proteins were conjugated with Iodine-125 (^125^I). The conjugated proteins included hen egg lysozyme (HEL) bovine colostrum and milk lactoferrin (BCL/BML), and bovine serum albumin (BSA). The iodine monochloride method [37,38] was used to radiolabel the proteins of interest, by covalently binding ^125^I to the tyrosine ring [39,40]. The radiolabeled proteins were added to the incubation solutions at a concentration of 2% of the individual protein concentration. Control solutions not containing a contact lens were used to verify radioactivity in the solution and decay over time.

### Single protein solution

The single protein solution (A) consisted of an augmented buffered saline solution containing different salts, glucose, and urea (Table 1). This was adapted from Van Haeringen [41] and further modified at the Centre for Contact Lens Research [42]. A single protein of interest was added to the solution.

**Table 1:** Components of the saline solution.

**Component**	**mM (mmol/ml)**	**MW (g/mol)**
C_6_H_12_O_6_*	0.2	180.2
CaCl_2_*	0.5	147
H_2_O	-	-
HCl (10 M)*	-	-
KCl^#^	16	74.55
KHCO*	3	100.12
Na_2_CO_3_^+^	12	105.99
Na_2_HPO_4_*	24	141.96
Na_3_C_6_H_5_O_7_^^^	1.5	294.1
NaCl*	90	58.44
(NH_2_)_2_CO^!^	1.2	60.06
ProClin 300*	-	-

C_6_H_12_O_6_ (glucose), CaCl_2_ (calcium chloride), H_2_O (Milli-Q gradient), HCl (10 M, hydrochloric acid), KCl (potassium chloride), KHCO_3_ (potassium bicarbonate), Na_2_CO_3_ (sodium carbonate), Na_2_HPO_4_ (sodium hydrogen phosphate), Na_3_C_6_H_5_O_7_ (trisodium citrate), NaCl (sodium chloride), (NH_2_)_2_CO (urea). * Sigma-Aldrich, Oakville, ON, ^#^ BDH Inc., Toronto, ON, ^+^ EMD Chemicals Inc., Gibbstown, NJ, ^^^ Caledon Laboratories LTD., Georgetown, ON, ^!^ EM Science, Gibbstown, NJ.

The saline solution was prepared with Milli-Q water in a glass beaker using a stir bar for constant mixing. The individual components were added to the solution. Finally, ProClin-300 (200 μl/l solution; Sigma, Oakville, ON), an antimicrobial agent, was added to the solution to inhibit bacterial growth. If necessary, NaOH was used to adjust the solution to a physiologic pH of 7.4 [43]. The pH was further determined at each study time point using pH paper (VWR, Mississauga, ON) to verify an unchanged environment of the solution at a pH of about 7 on a scale of 1–12.

The solution was split into three batches and one protein, either HEL (1.9 mg/ml), BCL/BML (1.9 mg/ml), or BSA (0.5 mg/ml) was added to make Solution A.

### Complex ATS

The complex ATS (B) consisted of the saline solution described above, plus proteins, lipids and mucins (Table 2). All lipids and proteins were purchased from Sigma-Aldrich.

**Table 2:** Components of the Complex Artificial Tear Solution.

**Lipids**	**C (mg/ml)**	**Proteins**	**C (mg/ml)**
Cholesterol [34-36]	0.0018	Albumin [44]	0.5
Cholesteryl oleate [34]	0.024	IgG [45-47]	0.02
Oleic acid [35,36]	0.0018	Lactoferrin [34,36]	1.8
Oleic acid methyl ester [34]	0.012	Lysozyme [34,36]	1.9
Phosphatidyl choline [48,49]	0.0005	Mucin [34,36]	0.15
Triolein [34]	0.016	Saline solution	-

The concentration of cholesterol was adapted through a formulation from a couple of studies. Haberland and colleagues [50] state in a study that the maximum solubility of cholesterol in aqueous solution is 0.0018 mg/ml. Interestingly, Saatci and colleagues [51] state that the concentration of cholesterol found in the tear film is greater. The IgG concentration was adapted from several literature values [45-47]. Coyle and Sibony [47] provide a range of IgG concentration found in the tear film that better relates to this study.

The saline solution was prepared as described above and all proteins were added (Table 2). Concentrated lipids were mixed in a separate flask with hexane-ether and sonicated for 5 min to break down the lipids into micelles. The lipid and protein solution were combined and nitrogen purged with nitrogen for 10 min to adjust the pH and evaporate hexane-ether.

### Contact lens incubation

All lenses were individually soaked in 5 ml of the prepared saline for 24 h, to remove any packaging solution components from the lenses. The lenses were handled with silicone-tipped tweezers in a sterile environment. Screw-capped glass vials (6 ml; VWR, Mississauga, ON) were autoclaved and pre-treated for 4–7 days with the same solution used for lens incubation, to coat the vials and minimize adsorption of elements to the walls of the vials during the lens incubation. During the pre-treatment phase, the concentration of lactoferrin (1.8 mg/ml) was halved to 0.9 mg/ml due to quantity and cost limitations. For similar reasons, both IgG and lactoferrin were omitted when pre-treating the complex ATS vials.

To fully submerge the lens, each lens was incubated in 1.5 ml of solution at 37 °C and placed on a rotatory shaker at 60 rpm (VWR). Time periods of 1, 7, 14, and 28 days were investigated using three replicates per lens type and time point, resulting in a total of 216 contact lenses being examined in the study.

After each incubation period, lenses were removed from the incubation solution, rinsed in saline twice, placed in a 12×75 mm culture tube (VWR), air-dried for 12 h to allow evaporation of unbound iodine. The Wallac Wizard 1470 Gamma Counter (Perkin Elmer, Woodbridge, ON) was used to quantify the amount of protein deposited on the lens.

## Results

This study consisted of two experiments, undertaken in parallel, to compare the deposition of lysozyme, lactoferrin and albumin to SH materials, when incubated in a single protein versus a complex ATS solution.

The pH of both solutions used for incubation (Solutions A and B) was checked at each time point. The results were in good agreement with the human tear film, which has a pH of approximately 7.4 [43]. Control solutions, not containing a contact lens, confirmed the anticipated amount of radioactivity in each solution, permitted us to monitor the radioactive decay over time and protein quantification.

Data analysis was conducted using Statistica 9 (StatSoft Inc. Tulsa, OK). A repeated measures ANOVA was used to compare protein deposition on the different lens materials over time. Factors included in the ANOVA were: protein of interest, contact lens material, and time point. Tukey’s HSD (Honestly Significant Difference) test was used for post-hoc comparisons; p<0.05 was considered significant.

### 

#### Lysozyme

**Figure 2:**
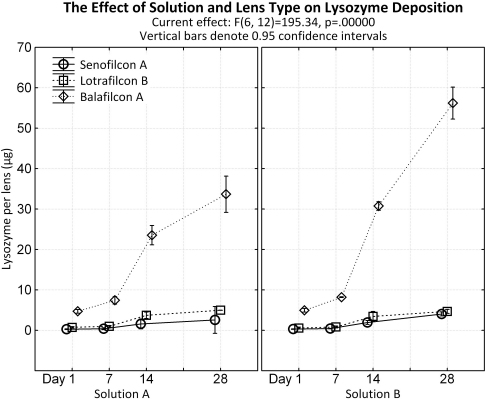
Lysozyme deposition to senofilcon A, lotrafilcon B, and balafilcon A lenses using a single protein solution and a complex ATS solution. Incubation time points: 1, 7, 14 and 28 days.

Results are presented in Figure 2. All lens types showed an increase in lysozyme deposition between days 1 and 28, independent of solution used for incubation (p<0.001).

After 1 day of incubation, SA lenses accumulated similar amounts of lysozyme when incubated in either solution (Solution A: 0.28±0.03 μg, Solution B: 0.31±0.03 μg; p=1.00). Slightly more lysozyme was found after 28 days: SA lenses accumulated 4.06±0.19 μg when incubated in Solution B and 2.57±1.33 μg using Solution A, however, this difference was also not statistically significant (p=0.20). LB lenses deposited similar amounts of lysozyme with both solutions (Solution A: 0.74±0.08 μg, Solution B: (0.58±0.09 μg); p=1.00) after 1 day of incubation. This amount increased after 28 days to 4.99±0.01 μg and 4.70±0.20 μg using Solution A and B, respectively (p=1.00). BA accumulated similar amounts of lysozyme after 1 day (Solution A: 4.69±0.19 μg, Solution B: 4.96±0.19 μg; p=1.00) independent of the solution used, but deposited significantly higher amounts after 14 and 28 days when incubated in Solution B (Day 28: Solution A=33.68±1.81 μg; Solution B=56.22±1.59 μg; p<0.001; Figure 2).

Overall, lysozyme deposition increased between each time point for either solutions (A and B) over a period of 28 days (p<0.001), with Solution B depositing significantly more lysozyme than Solution A by day 28 (p<0.001). Independent of lens type, lysozyme deposition increased from day 1 to 28, depositing significantly greater amounts of protein between each time point, for both Solutions A and B (p<0.001).

#### Lactoferrin

**Figure 3:**
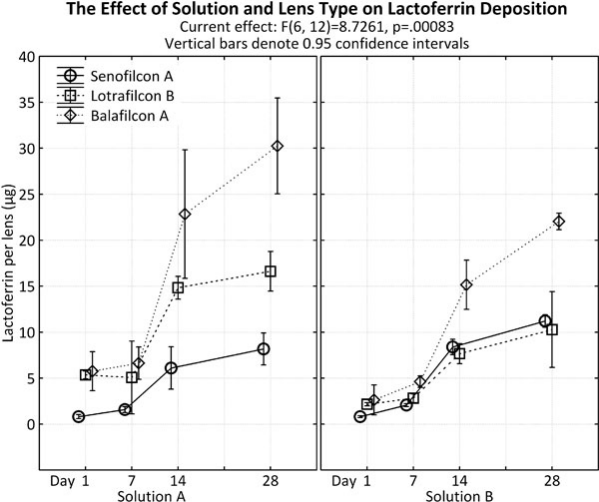
Lactoferrin deposition to senofilcon A, lotrafilcon B, and balafilcon A lenses using a single protein solution and a complex ATS solution. Incubation time points: 1, 7, 14 and 28 days.

Results are presented in Figure 3. From day 1 to 28, the amount of lactoferrin deposition for each lens type increased independent of solution used for incubation (p<0.001).

After 1 day of incubation, SA lenses accumulated similar amounts of lactoferrin using Solution A (0.81±0.09 μg) and Solution B (0.81±0.04 μg; p=1.00). After 28 days, slightly less lactoferrin was seen when incubated in Solution A (8.17±0.70 μg) in comparison to Solution B (11.21±0.28 μg; p=0.17). LB lenses incubated in Solution A (5.34±0.17 μg) accumulated slightly more lactoferrin than Solution B (2.16±0.07 μg) after 1 day, however this difference was not significant (p=0.13). After 28 days however, LB deposited significantly more lactoferrin when incubated in Solution A compared to Solution B (Solution A: 16.62±0.86 μg, Solution B: 10.28±1.66 μg; p<0.001). BA lenses also attracted slightly higher amounts of lactoferrin when incubated in Solution A (5.75±0.86 μg) compared to Solution B (2.62±0.66 μg) after 1 day (p=0.14) which became statistically significant after 28 days, where Solution A allowed for 30.25±2.10 μg of deposits on the lenses compared to Solution B (22.04±0.51 μg; p<0.001; Figure 3).

There was overall an increase in lactoferrin deposition between days 1 and 28 (p<0.001), with Solution A depositing significantly more lactoferrin than Solution B (p=0.017). Independent of lens type, lactoferrin deposits similarly on lens materials incubated in Solution A at day 1 and 7 (p=0.91). However, there was an increase in the rate of accumulation between the other time points (day 7 and 14 [p<0.001]; day 14 and 28 [p=0.001]). With regards to Solution B, there was not a significant difference between lactoferrin deposits at days 1 and 7 (p=0.52), 14 and 28 (p=1.00), but a significant difference between days 7 and 14 (p<0.001).

#### Albumin

**Figure 4:**
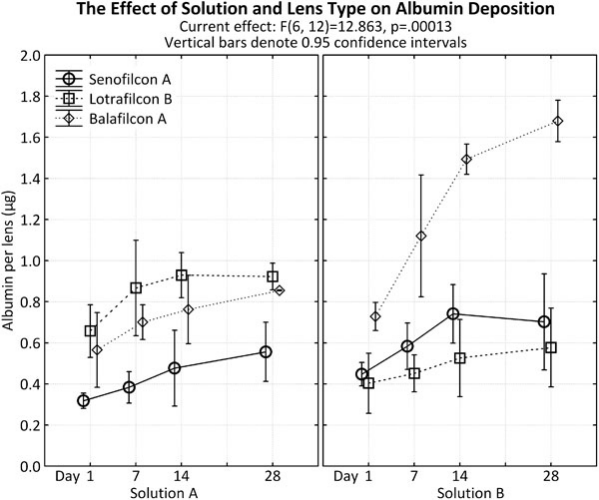
Albumin deposition to senofilcon A, lotrafilcon B, and balafilcon A lenses using a single protein solution and a complex ATS solution. Incubation time points: 1, 7, 14 and 28 days.

Results are presented in Figure 4. In general, there was an increase in albumin deposition for each lens type from day 1 to 28, independent of solution used for incubation (p<0.001).

After 1 day of incubation, SA lenses accumulated similar amounts of albumin when incubated in either solution (Solution A: 0.32±0.02 μg, Solution B: 0.45±0.02 μg; p=0.70). A similar result was also seen after 28 days, showing 0.56±0.06 μg when incubated in Solution A and 0.70±0.09 μg using Solution B (p=0.53). LB deposited significantly more albumin in Solution A (0.66±0.05 μg) compared to Solution B (0.40±0.06 μg) after 1 day of incubation (p=0.04). Likewise more albumin was accumulated after 28 days when lenses were incubated in Solution A (0.92±0.03 μg) compared to Solution B (0.58±0.08 μg; p=0.003). After 1 day of incubation, BA lenses deposited similar amounts of albumin with both solutions (Solution A [0.57±0.07 μg]; Solution B [0.73±0.03μg; p=0.38]). With a longer incubation of 28 days, BA lenses accumulated significantly less (p<0.001) albumin in Solution A (0.85±0.00 μg) in comparison to Solution B (1.68±0.04 μg; Figure 4).

Overall, there was an increase in albumin deposition between day 1 and day 28 and also between each time point (p<0.001), with Solution B depositing significantly more albumin than Solution A (p=0.008). Independent of lens type, the amount of albumin increased from day 1 to day 7 (p=0.01) using Solution A and a plateau was seen after this time point (day 7 and 14 [p=0.18], day 14 and 28 [p=0.40]). With regards to Solution B, there was a significant difference between each time point, as protein deposition increased significantly at each measurement; day 1 and 7 (p=0.02), day 7 and 14 (p<0.001), day 14 and 28 (p<0.001).

## Discussion

The human tear film contains a variety of proteins, lipids, and mucins, each of which differs in size, charge, and concentration [29,52,53]. Positively charged lysozyme [26] (14.5 kDa [54]) and the iron-binding protein lactoferrin (80 kDa [55]) constitute major proportions of the tear film, measuring 1.9 mg/ml [56] and 1.5–2.2 mg/ml [55], respectively. Lysozyme contains three positive binding sites [57], whereas lactoferrin has one [58]. Albumin, a negatively charged protein, has a molecular weight of 66 kDa [59] and is found in the tear film at a concentration ranging from 0.02 to 0.5 mg/ml [60,61].

The contact lens materials investigated in this study were incubated in a non-competitive, single protein solution and a complex ATS, consisting of multiple proteins, mucins, and lipids. This complex ATS, according to the Vroman effect, will allow for sequential adsorption of proteins to the lens surface [52,62,63]. Blood plasma proteins undergo the Vroman effect when adsorbing onto artificial surfaces, particularly the displacement of fibrinogen by other plasma proteins [64]. Sariri and Sabbaghzadeh [65] have demonstrated competitive protein binding onto soft contact lens surfaces and the ability of proteins to displace one another. To-date, only a few studies have determined the impact of other tear components during the sorption process, and no data were available on proteins of different charge, size and abundance and their interaction with SH materials [25,30]. It was predicted that the negatively charged albumin would deposit to only a minor extent onto negatively charged materials, due to electrostatic repulsion [66].

The three SH lenses investigated in this study differed in material composition, water content and surface modification. The SA material contains a copolymerization of HEMA and N, N-dimethyl acrylamide with (3-methylacryloxy-2hydroxypropyloxy) propylbis (trimethylsiloxy) methylsilane [67]. In addition, an internal wetting agent (polyvinyl pyrrolidone (PVP)) is incorporated into SA lenses to improve wettability [68-70]. Lysozyme contributes 6–13 μg of the total protein deposition per SA lens in in vitro studies [27,30], whereas ex vivo studies report up to 7 μg of total protein per lens deposited [5,28,33], with lysozyme contributing about 25% [28,33], demonstrating that more lysozyme is deposited in vitro (6–13 μg versus 1.75 μg). For SA lenses, there was no statistically significant difference in deposition of any of the proteins investigated whether Solution A or B was used for incubation (p=NS). This suggests that this material is unaffected by incubation solution composition. Given the complex nature of the ATS, this result suggests that little competition for protein deposition occurs with this material, and that protein deposition is driven by non-competitive factors. After 28 days, SA also deposited the lowest amount of all three proteins, as compared with the other two materials. This low level of deposition has been seen in other in vitro and ex vivo studies [27,28,30,33], and may be attributed to the neutral surface charge and, specifically, the presence of PVP, which for both contact lenses and other biomaterial applications has also been shown to exhibit low levels of protein deposition [30,71].

The LB material has a co-continuous biphasic- siloxy and hydrogel phase, which aids the lens in maintaining oxygen and salt transmission [67]. This lens material is coated by hydrophilic plasma to improve hydrophilicity of the surface [67,72] and this plasma coating (25 nm thick) limits access to the underlying polymer, hence decreasing protein deposition on this material and within the matrix [6,73]. In vitro studies on LB show that lysozyme contributes about 6–10 μg of total protein deposited per lens [25,27]. Ex vivo studies illustrate that >7 μg [74,75] of total protein per lens is deposited, with <25% being lysozyme [28,33]. After 28 days of incubation, no differences in the amounts of lysozyme deposited on LB lenses were measured between the two incubation solutions (p=NS). This may be due to the size of lysozyme, which is the smallest of the three proteins and may outcompete the other two proteins, appearing as if it is accumulating on the lens material without competition from other proteins. A significant difference in both lactoferrin and albumin accumulation occurred (p<0.05), with the simplistic incubation solution (Solution A) producing the greatest deposition. These data suggest that when exposed to Solution A, which has no lysozyme, the other two proteins of interest can deposit freely, without the competitive binding that lysozyme exhibits. After 28 days, in comparison with the other two materials, LB deposits more protein than SA, but less than BA.

The BA material has a biphasic character due to copolymerization of the TRIS derivative vinyl carbamate and N-vinyl pyrrolidone [67]. Hydrophilic glassy silicate ‘islands’ can be seen on the surface of BA lenses [6] due to the oxidation of TRIS [67]. BA is considered ionic (FDA Group III) due to its incorporation of N-vinyl aminobutyric acid and as a result, this material typically accumulates more tear proteins, particularly those that are positively charged, compared to other SH lenses [5,27,30]. Furthermore, unlike other SH lenses, the surface of BA is more porous, allowing for protein to penetrate through the matrix [72,73]. Of the total amount of protein depositing on worn BA lenses (5–34 μg) [5,33], lysozyme accounts for 32% [76] to 50% [25]. Previous in vitro studies report that lysozyme deposits approximately 10-20 μg of protein per lens [30,76]. Lysozyme accumulated significantly more on BA lenses (p<0.05) when incubated in the complex ATS. This is an interesting phenomenon, as it would be predicted that there would be no difference between the two solutions because of lysozyme’s ability to deposit on a negatively charged material in large amounts, independent of incubation solution. One potential explanation could be that when exposed to a complex ATS that there is an initial deposition of the positively charged lysozyme, which acts to partially neutralize the surface charge of the BA material, allowing some binding of the negatively charged albumin, which then results in a “layering” of proteins on top of this initial layer [77,78]. Lactoferrin, as expected, deposited significantly more on the BA material when incubated in the simplistic solution. This is due to the decrease in available binding sites on the negatively charged BA material, due to lysozyme’s competitive behavior. In contrast, albumin deposited more when lenses were incubated in the complex ATS (Solution B). The low level when exposed to Solution A is expected, as both BA and albumin are negatively charged and exhibit mutual electrostatic repulsion. The higher level when exposed to the complex Solution B can be attributed to the partial neutralization of the BA material by the positively charged lysozyme and lactoferrin, allowing albumin for an increased opportunity to bind to the BA surface. Of the three materials examined, BA deposits the highest amount of all three proteins.

Patient-worn senofilcon A lenses deposit approximately 7 μg [28,33] of total protein, whereas lenses incubated in Solution A and B deposited approximately two times more protein (11.30 μg and 15.97 μg, respectively (sum of ^125^I data from all three proteins). Lotrafilcon B lenses in Solution A deposited approximately three times more total protein (22.53 μg) than what has been found in ex vivo studies (>7 μg [14,74,75]), whereas Solution B lenses accumulated roughly two times more total protein (15.56 μg). Ex vivo studies on balafilcon A have found 5–34 μg [5,33] of total protein, whereas BA lenses incubated in Solution A accumulated two times more protein (64.78 μg) versus approximately three times more total protein when using Solution B (79.94 μg). Several reasons may account for these differences. The naturally occurring physiologic events of the eye, blinking, and surface drying are all lacking in this in vitro model. The lens surface in vivo dries between blinks as the lid wipes over the material [79] and this drying is known to influence deposition onto lens materials from the tear film [80]. Most importantly, ex vivo studies typically contain the use of a care regimen each day, which would be predicted to decrease protein accumulation on the lens material over time.

In conclusion, this study confirms that there are differences in amounts of protein deposition onto SH materials incubated in either a single protein or complex ATS incubation solution. The results showed that protein accumulation was further dependent on incubation time, the nature of the protein (size, concentration, and charge) and type of SH material. BA was the greatest accumulator, as previously reported. With regards to lysozyme deposition, no impact of the type of solution was seen for SA and LB lenses, however, BA lenses incubated in Solution B deposited greater amounts of lysozyme. Greater amounts of lactoferrin also accumulated on LB and BA lenses when incubated in Solution A, whereas the opposite trend was seen for SA lenses, which deposited more lactoferrin with Solution B. Finally, BA lenses deposited greater amounts of albumin when incubated in Solution B, whereas LB lenses accumulated more albumin when incubated in Solution A, while less solution impact was found using SA lenses.

The diversity of the results in this study highlights the importance of using appropriate in vitro models, as the outcome for protein accumulation to certain contact lens - protein interactions is strongly impacted by the competitive nature of the respective tear film components.
